# Adipose Tissue Browning in MASLD and Its Molecular Mechanisms and Metabolic Crosstalk

**DOI:** 10.1007/s13679-026-00746-z

**Published:** 2026-08-01

**Authors:** Jonathan Jaime G. Guerrero, Mark Angelo S. del Rosario, Paolo C. Encarnacion, Chen-Sung Lin, Lu-Te Chuang, Kin Israel Notarte, Jiayan Zhou, Chih-Hao Wang, Ching-Wen Chang, Wan-Chun Li

**Affiliations:** 1 College of Medicine, University of the Philippines Manila, Manila, Philippines; 2 College of Public Health, University of the Philippines Manila, Manila, Philippines; 3 Division of Thoracic Surgery, Department of Surgery, Taipei Hospital, Ministry of Health and Welfare, New Taipei City, Taiwan; 4 School of Medicine, College of Medicine, National Yang Ming Chiao Tung University, Taipei, Taiwan; 5 Center for General Education, Kainan University, Taoyuan City, Taiwan; 6 Department of Biotechnology and Pharmaceutical Technology, Yuanpei University of Medical Technology, Hsinchu, Taiwan; 7 Department of Pathology, Johns Hopkins University School of Medicine, Baltimore, MD USA; 8 Department of Medicine, Stanford University School of Medicine, Stanford, CA USA; 9 Genomics Research Center, Academia Sinica, Taipei, Taiwan; 10 Graduate Institute of Metabolism and Obesity Sciences, Taipei Medical University, Taipei, Taiwan; 11 TMU Research Center for Digestive Medicine, Taipei Medical University, Taipei, Taiwan; 12 Taipei Cancer Center, Taipei Medical University, Taipei, Taiwan; 13 Institute of Oral Biology, College of Dentistry, National Yang Ming Chiao Tung University, Taipei, Taiwan; 14 Department of Dentistry, College of Dentistry, National Yang Ming Chiao Tung University, Taipei, Taiwan; 15 Oral Medicine Innovation Center (OMIC), National Yang Ming Chiao Tung University, Taipei, Taiwan; 16 Department of Stomatology, Taipei Veterans General Hospital, Taipei, Taiwan

**Keywords:** Adipose tissue, Browning, Beige adipocytes, MASLD, Liver metabolism

## Abstract

**Purpose of Review:**

Metabolic dysfunction–associated steatotic liver disease (MASLD) is an increasingly prevalent complication of obesity and metabolic dysregulation, with limited therapies targeting upstream drivers of disease. Adipose tissue has emerged as a central regulator of systemic metabolic homeostasis, where dysfunction contributes to excess free fatty acid flux, chronic inflammation, and hepatic steatosis. In this context, adipose tissue browning—the induction of thermogenically active beige adipocytes within white adipose depots—has gained attention as a potential therapeutic mechanism.

**Recent Findings:**

Recent advances highlight that adipose browning modulates multiple pathways relevant to MASLD. These include enhanced mitochondrial β-oxidation and energy expenditure, leading to reduced lipid delivery to the liver, as well as endocrine signaling mediated by batokines such as fibroblast growth factor 21 (FGF21), irisin, and neuregulin 4 (Nrg4). Collectively, these pathways influence hepatic lipid metabolism, insulin sensitivity, and inflammatory and fibrotic processes. The preclinical studies consistently demonstrate metabolic and hepatoprotective benefits of browning; however, translational evidence in humans remains limited and heterogeneous. Factors such as reduced thermogenic capacity in obesity, inter-individual variability, and challenges in sustaining browning activation constrain clinical applicability.

**Summary:**

Overall, adipose tissue browning represents a promising component of a systems-based approach to MASLD. Future work should focus on integrating mechanistic insights with clinical investigation to clarify its therapeutic potential and to identify strategies that enable durable and patient-specific metabolic benefits.

**Graphical Abstract:**

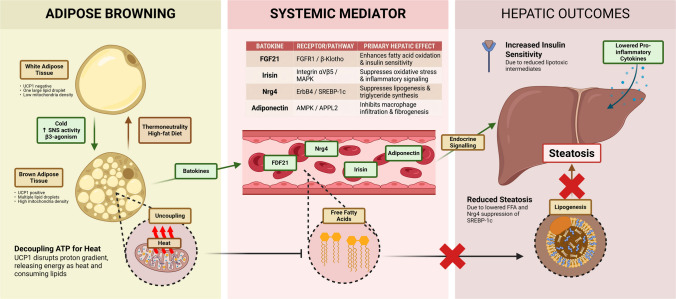

## Introduction

Metabolic dysfunction–associated steatotic liver disease (MASLD) has become one of the most prevalent chronic liver diseases worldwide, affecting nearly one-third of the global adult population [1, 2] and rising in parallel with the epidemics of obesity and type 2 diabetes [3, 4]. Its burden is region-specific [5], affected by rapid nutritional and lifestyle transitions [6]. MASLD encompasses a progressive spectrum from simple steatosis to metabolic dysfunction–associated steatohepatitis [MASH, historically termed nonalcoholic steatohepatitis (NASH)], advanced fibrosis, and cirrhosis, and is now a leading indication for liver transplantation and a major contributor to hepatocellular carcinoma [7–9]. Beyond liver-related outcomes, MASLD confers substantial cardiovascular risk [10, 11], reinforcing its identity as a systemic metabolic disorder. Despite its scale and impact, therapeutic options remain limited, with most current interventions targeting downstream hepatic injury rather than the upstream metabolic disturbances that drive disease onset and progression.

Central to this upstream pathology is adipose tissue, which has emerged as a dynamic endocrine and metabolic organ rather than a passive reservoir of energy [12, 13]. In metabolically unhealthy states, dysfunctional white adipose tissue promotes increased lipolysis, insulin resistance, and chronic low-grade inflammation, resulting in an excess flux of free fatty acids to the liver and subsequent lipotoxicity [13, 14]. In contrast, adipose tissue retains a capacity for phenotypic remodeling through “browning,” wherein white adipocytes acquire beige or brown-like characteristics [15]. This process is governed by key molecular regulators, including uncoupling protein 1 (UCP1), PR domain containing 16 (PRDM16), and PGC-1α, which together drive mitochondrial biogenesis, thermogenesis, and enhanced fatty acid oxidation [16]. These mechanisms have been described in literature [17, 18]. By increasing energy expenditure and improving systemic metabolic efficiency, adipose browning may be a biologically compelling counter-regulatory mechanism to the metabolic drivers of MASLD [19].

Importantly, the relevance of adipose browning extends beyond energy dissipation to encompass inter-organ communication. Browned adipose tissue secretes a repertoire of signaling molecules called batokines such as fibroblast growth factor 21 (FGF21), irisin, neuregulin 4 (Nrg4), and 12,13-diHOME, which influence hepatic lipid handling, glucose metabolism, and inflammatory signaling pathways [20–22]. These observations support the existence of a tightly regulated adipose–liver axis, wherein alterations in adipose tissue phenotype can directly modulate hepatic physiology [23]. However, despite increasing recognition of this axis, the gaps remain and mechanistic insights dispersed across literature.

Adipose tissue is a heterogeneous organ composed of multiple depots with distinct anatomical, metabolic, and endocrine properties, which differ substantially between species [24, 25]. In murine models, white adipose tissue (WAT) depots are commonly divided into epididymal WAT (eWAT), a visceral depot with high lipolytic activity and strong association with metabolic dysfunction, and inguinal WAT (iWAT), a subcutaneous depot with a greater propensity for browning and thermogenic remodeling [24, 26]. Classical brown adipose tissue (BAT), primarily located in the interscapular region in mice, exhibits high mitochondrial density and constitutive thermogenic activity [20, 27].

In humans, adipose depots are typically categorized into visceral adipose tissue (VAT), which drains into the portal circulation and contributes directly to hepatic lipid flux and inflammation, and subcutaneous adipose tissue (SAT), which serves as a metabolic buffer and exhibits limited but inducible browning capacity [25, 26]. Human BAT is predominantly located in cervical and supraclavicular regions and displays substantial inter-individual variability, with lower overall mass and thermogenic contribution compared to rodents [28, 29]. Importantly, depot-specific differences in browning capacity, lipolysis, inflammatory profile, and anatomical connectivity to the liver have major implications for MASLD pathophysiology and for translating findings from murine models to human disease [20, 30].

Given the central role of adipose tissue in driving metabolic dysregulation in MASLD, it is important to distinguish between adipose depots and species-specific differences in their biological function. Adipose tissues are not homogeneous; rather, they exhibit marked variability in browning capacity, metabolic activity, inflammatory profile, and anatomical connection to the liver. These differences have important implications for interpreting mechanistic studies and translating findings from animal models to human disease. Table 1 summarizes key differences between adipose depots across species, highlighting variations in browning capacity, metabolic function, inflammatory profile, and anatomical linkage to the portal circulation, based on published literature and experimental studies on adipose tissue heterogeneity, thermogenic adipocytes, and MASLD pathophysiology [24, 25, 30–32]. These depot-specific and species-specific distinctions are essential for understanding the adipose–liver axis in MASLD. In particular, visceral depots with direct portal drainage exert a disproportionate influence on hepatic lipid flux and inflammation, whereas subcutaneous and thermogenic depots primarily modulate systemic metabolism through energy expenditure and endocrine signaling.

**Table 1:** Depot-specific and species-specific characteristics of adipose tissue relevant to browning and MASLD pathophysiology

Depot Type	Species	Anatomical Location	Browning Capacity	Primary Metabolic Role	Portal Connection	Inflammatory Profile	Contribution to MASLD	Evidence Type
eWAT (epididymal WAT)	Mouse	Visceral (intra-abdominal)	Low	Lipid storage; high lipolysis	Yes (portal drainage)	High (pro-inflammatory)	Major contributor to hepatic FFA flux and lipotoxicity	Preclinical
iWAT (inguinal WAT)	Mouse	Subcutaneous	High (beige induction)	Thermogenesis (inducible) + storage	No	Low to moderate	Limited direct contribution; indirect metabolic benefits via browning	Preclinical
Classical BAT (interscapular)	Mouse	Interscapular region	Very high	Thermogenesis (UCP1-driven)	No	Low	Reduces systemic lipid availability; indirect hepatoprotection	Preclinical
VAT (visceral adipose tissue)	Human	Omental/mesenteric	Very low	Lipid storage; high lipolysis	Yes (direct portal vein)	High (pro-inflammatory cytokines)	Major driver of hepatic steatosis, inflammation, and MASH progression	Clinical + epidemiologic
SAT (subcutaneous adipose tissue)	Human	Peripheral (e.g., abdominal, femoral)	Moderate (beige potential)	Lipid storage; metabolic buffering	No	Lower than VAT	Protective role; browning may reduce systemic lipid burden	Clinical + experimental
BAT (supraclavicular/cervical)	Human	Cervical, supraclavicular	Variable (age- and BMI-dependent)	Thermogenesis; endocrine signaling	No	Low	Associated with improved metabolic profile but unclear causal role in MASLD	Imaging + observational
Beige adipocytes (inducible)	Mouse/Human	Primarily in subcutaneous depots	Inducible	Thermogenesis under stimuli (e.g., cold, exercise)	No	Low	Potential indirect role in reducing hepatic lipid load and improving insulin sensitivity	Preclinical (strong), human (limited)

This fragmentation underscores a critical gap in the current literature and highlights the need for a comprehensive synthesis of mechanistic evidence. A key question remains: to what extent can adipose tissue browning be leveraged to influence the development and progression of MASLD, and what are its net effects on hepatic steatosis, inflammation, and fibrogenesis across disease stages? Addressing this question requires a deeper understanding of the molecular mediators governing adipose–liver crosstalk, the feasibility and durability of inducing browning in humans, and the extent to which these mechanisms translate into clinically meaningful outcomes. In this context, the present review aims to consolidate functional outcomes of adipose tissue browning in MASLD and identify key knowledge gaps that must be addressed to advance this promising field toward clinical and public health applications.

## Methodology

This narrative review summarizes current evidence regarding adipose tissue browning and its role in MASLD. Relevant literature was identified through searches of PubMed, Scopus, and Web of Science using combinations of terms related to adipose tissue browning, brown adipose tissue, beige adipocytes, batokines, adipose–liver crosstalk, MASLD, and MASH. Priority was given to recent mechanistic studies, translational investigations, clinical trials, and high-quality review articles to provide a comprehensive overview of current knowledge and emerging therapeutic directions.

## Mechanistic Crosstalk Between Adipose Browning and Hepatic Metabolism in MASLD

To better conceptualize the complex interactions between adipose tissue and the liver in MASLD, the mechanistic pathways described below can be reorganized into an integrated adipose tissue–liver axis. This axis comprises three interconnected components: (i) adipose-to-liver signaling, (ii) liver-to-adipose signaling, and (iii) bidirectional feedback loops that coordinate systemic metabolic homeostasis. Framing adipose browning within this axis-based model allows for a more coherent understanding of how thermogenic activation influences hepatic steatosis, inflammation, and fibrogenesis across disease stages.

### Adipose → Liver Axis: Regulation of Hepatic Substrate Load and Lipotoxicity

Dysfunctional adipose tissue is a primary upstream driver of hepatic steatosis in MASLD. In insulin-resistant states, enhanced lipolysis in white adipose tissue—particularly visceral depots—increases circulating and portal free fatty acids (FFAs) flux, directly delivering substrate to the liver. This excessive lipid influx promotes triglyceride accumulation and formation of lipotoxic intermediates, contributing to hepatic insulin resistance, inflammation, and disease progression. Adipose tissue browning modifies this axis by shifting adipocytes toward a lipid-consuming phenotype characterized by enhanced mitochondrial β-oxidation and UCP1-mediated thermogenesis. By reducing net FFA efflux and circulating lipid availability, browning decreases hepatic substrate burden and may attenuate early steatosis. At the level of MASLD progression, this axis primarily influences the transition from steatosis to early metabolic dysfunction, with indirect effects on downstream inflammatory and fibrotic pathways.

#### Adipose Lipid Flux Regulation

**Fig. 1:**
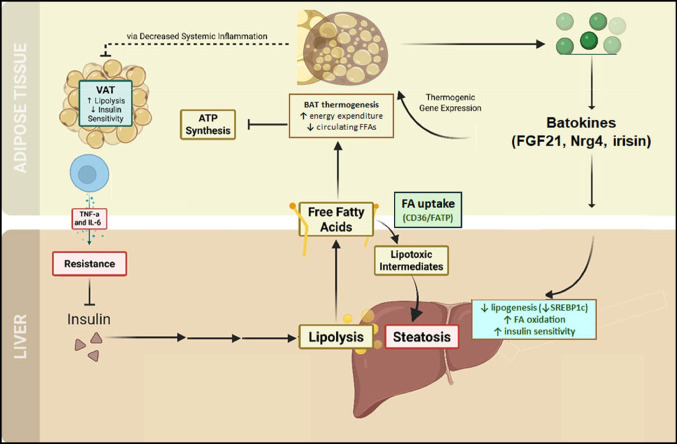
Adipose–liver metabolic interplay in MASLD: regulation of lipid flux, inflammation, and thermogenic activation. Schematic overview of the adipose–liver axis in MASLD, highlighting how adipose tissue dysfunction and thermogenic activation regulate hepatic lipid metabolism. In insulin-resistant visceral adipose tissue (VAT), enhanced lipolysis and impaired insulin signaling increase systemic and portal free fatty acid (FFA) flux, accompanied by pro-inflammatory cytokines (e.g., TNF-α and IL-6). These factors promote hepatic FFAs uptake via transporters such as CD36 and FATPs, leading to accumulation of lipotoxic intermediates (e.g., diacylglycerols, ceramides) and steatosis. Conversely, activation of brown and beige adipocytes enhances mitochondrial β‑oxidation and dissipates energy via UCP1-mediated thermogenesis, thereby reducing circulation of FFA availability. In addition, BAT-derived endocrine factors (batokines), including FGF21, Nrg4 and irisin, modulate hepatic metabolism by suppressing lipogenesis and promoting fatty acid oxidation. Importantly, while these pathways are strongly supported in preclinical models, their quantitative contribution in human MASLD may be attenuated by variability in brown adipose tissue (BAT) mass and activity, particularly in obesity. Several pathways depicted in this figure are primarily supported by preclinical studies, particularly murine models of adipose browning and MASLD. While key mechanisms such as FGF21 signaling, adipose–liver lipid flux, and BAT-associated metabolic regulation have supporting human data, other pathways remain incompletely validated in clinical settings. Therefore, the strength of evidence varies across the illustrated interactions, and direct translation to human MASLD should be interpreted cautiously. The figure was created with BioRender.com; a publication license was obtained

Adipose tissue is a primary regulator of systemic lipid turnover [33] and therefore plays a critical role in determining hepatic substrate delivery in MASLD. Under physiological conditions, insulin suppresses triglyceride hydrolysis by inhibiting adipose triglyceride lipase (ATGL) [34] and hormone-sensitive lipase (HSL) [35], primarily through activation of the insulin receptor substrate–phosphoinositide 3-kinase–protein kinase B (IRS–PI3K–AKT) signaling cascade [36]. In insulin-resistant states, however, this pathway is impaired, resulting in sustained lipolysis and continuous release of FFAs into the circulation [37]. Elevated circulating FFAs are subsequently taken up by hepatocytes, where they are directed toward triglyceride synthesis or converted into bioactive lipotoxic intermediates such as diacylglycerols (DAGs) and ceramides and long-chain acylcarnitines [38]. These intermediates disrupt hepatic insulin signaling and contribute to steatosis and metabolic dysfunction [39, 40]. Thus, dysregulated adipose tissue lipolysis represents a major upstream driver of hepatic lipid overload in MASLD. From a quantitative perspective, hepatic triglyceride accumulation in MASLD is derived from multiple sources, including adipose tissue–derived non-esterified fatty acids (NEFAs), hepatic de novo lipogenesis, and dietary lipids. Among these, adipose-derived NEFAs represent the dominant contributor under insulin-resistant conditions, highlighting the central role of adipose tissue dysfunction in driving hepatic steatosis within the adipose–liver axis. In contrast, increased de novo lipogenesis further amplifies lipid accumulation and becomes more prominent with disease progression. This quantitative hierarchy reinforces the central role of adipose tissue as the primary upstream driver of hepatic lipid overload in early MASLD, while highlighting the increasing contribution of intrahepatic metabolic dysregulation in later disease stages. Figure 1 illustrates the adipose tissue–liver axis, highlighting unidirectional (adipose-to-liver and liver-to-adipose) and bidirectional feedback mechanisms that regulate lipid flux, thermogenesis, endocrine signaling, and MASLD progression. This framework provides a foundation for understanding how upstream adipose dysfunction translates into hepatic metabolic pathology.

Adipose tissue browning modifies this pathological flux by enhancing intracellular fatty acid utilization. Thermogenic adipocytes increase mitochondrial β-oxidation and dissipate energy through uncoupling protein 1 (UCP1), thereby reducing net FFA efflux into the circulation [41, 42]. In this context, brown and beige adipocytes function as a metabolic sink, shifting adipose tissue from a lipid-exporting to a lipid-consuming phenotype. This reduction in circulating FFAs limits hepatic substrate availability and may attenuate lipotoxic stress.

At a systems level, this shift highlights a key axis in MASLD pathophysiology: the balance between lipid release from dysfunctional white adipose tissue and lipid clearance by thermogenically active depots. While this mechanism is well supported in preclinical models, its magnitude and consistency in human metabolic disease remain less clearly defined. This mechanism represents a central component of the adipose-to-liver axis, whereby dysregulated adipose lipolysis directly determines hepatic substrate availability. It predominantly contributes to early disease stages, driving hepatic steatosis and initiating metabolic dysfunction that may progress to MASH.

#### Hormonal Regulation of Adipose Lipolysis

Adipocyte lipolysis is tightly regulated by the opposing actions of insulin and catecholamines, which converge on intracellular signaling pathways controling lipase activity. Insulin exerts a strong antilipolytic effects by activating phosphodiesterase 3B (PDE3B) [43], leading to degradation of cyclic adenosine monophosphate (cAMP) and suppression of protein kinase A (PKA)-mediated phosphorylation of HSL and perilipin [43, 44]. In insulin-resistance states, impairment of IRS–PI3K–AKT signaling diminishes this inhibitory effect, allowing lipolysis to proceed unchecked [44].

In contrast, catecholamines stimulate lipolysis through β-adrenergic receptor activation, particularly via β3-adrenergic receptors in adipocytes [45]. This signaling increases intracellular cAMP levels, activates PKA, and promotes phosphorylation of lipolytic enzymes. Chronic sympathetic activation, frequently observed in obesity, sustains this pathway and contributes to elevated circulating FFAs [46]. Additional modulators, including glucocorticoids and natriuretic peptides, further influence lipolysis through cyclic guanosine monophosphate (cGMP)-dependent signaling pathways, underscoring the complexity of hormonal regulation in adipose tissue.

In thermogenic adipocytes, however, lipolysis is closely coupled to mitochondrial oxidation. Fatty acids released in response to catecholamine signaling are rapidly oxidized within mitochondria, preventing excessive systemic release. Concurrent improvements in insulin sensitivity partially restore antilipolytic control. This coordinated regulation suggests that browning does not simply increase lipolysis, but rather reprograms lipid turnover toward efficient utilization, thereby reducing the net contribution of adipose tissue to circulating lipid burden.

#### Visceral Adipose Tissue as a Major Source of Portal FFAs

Visceral adipose tissue (VAT) plays a particularly important role in hepatic lipid accumulation due to its direct anatomical connection to the liver via the portal circulation. FFAs released from VAT enter the portal vein and are delivered directly to hepatocytes, resulting in high local substrate concentrations that favor triglyceride synthesis and impair insulin signaling [47].

Compared with subcutaneous depots, VAT exhibits enhanced lipolytic activity, characterized by increased expression of ATGL and HSL, reduced insulin responsiveness, and heightened β-adrenergic signaling [48, 49]. Additionally, VAT is enriched in pro-inflammatory immune cells that secrete cytokines such as tumor necrosis factor-alpha (TNF-α) and interleukin-6 (IL-6) [50, 51], further promoting lipolysis and exacerbating insulin resistance.

Although adipose browning predominantly occurs in subcutaneous depots, its systemic metabolic effects can indirectly influence VAT function. By reducing circulating FFAs and improving overall insulin sensitivity, browning may suppress VAT lipolysis [50] and decrease portal lipid delivery. This indirect modulation highlights an important distinction: browning does not necessarily target the most pathogenic depot directly but could still improve its downstream metabolic consequences. Within the adipose-to-liver axis, VAT serves as a critical upstream source of portal FFA flux, exerting a disproportionate effect on hepatic lipid accumulation and inflammation. This mechanism is particularly relevant to the transition from simple steatosis to inflammatory MASH due to its combined metabolic and pro-inflammatory impact.

#### Lipid Transport Mechanisms

Hepatic uptake of circulating FFAs is mediated by membrane-associated transporters, including cluster of differentiation 36 (CD36) and fatty acid transport proteins (FATPs), particularly FATP2 and FATP5 [52, 53]. Upregulation of these transporters enhances hepatocellular fatty acid uptake, contributing to intracellular lipid accumulation and lipotoxic stress [54–56]. In circulation, FFAs are primarily bound to albumin, and their distribution is determined by concentration gradients, tissue perfusion, and transporter expression [57]. Elevated plasma FFA levels therefore directly increase hepatic uptake, especially under conditions where transporter expression is upregulated, reinforcing substrate-driven steatosis [58].

Adipose browning reduces circulating FFA concentrations by increasing fatty acid uptake and oxidation within thermogenic adipocytes [59]. This not only lowers substrate availability for hepatic transporters but may also secondarily reduce transporter expression under improved metabolic conditions. Together, these effects contribute to limiting hepatic lipid accumulation. Importantly, while these mechanisms provide a coherent framework linking adipose tissue activity to hepatic lipid handling, their quantitative contribution in human MASLD remains an area of ongoing investigation. This process represents a downstream component of the adipose-to-liver axis, translating circulating lipid availability into hepatocellular lipid accumulation. It primarily influences steatosis progression and amplifies lipotoxic signaling that contributes to inflammation and early fibrogenesis.

**Fig. 2:**
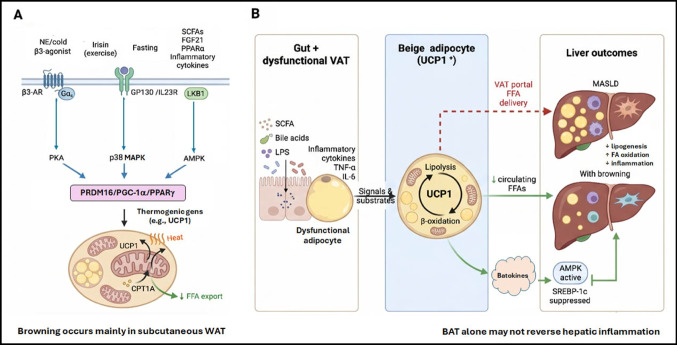
Molecular signaling pathways and inter-organ crosstalk driving adipose tissue browning in MASLD. **A** Core intracellular pathways regulate adipose browning. External stimuli—including cold exposure, sympathetic activation (β3-adrenergic signaling), exercise-derived irisin, fasting, and metabolic signals [(e.g. short-chain fatty acids (SCFAs) and FGF21]—activate convergent signaling cascades such as β3‑adrenergic receptor–cAMP–PKA, AMPK, and p38 MAPK pathways. These pathways regulate key transcriptional programs mediated by PRDM16, PGC-1α, and PPARγ, leading to induction of thermogenic gene (UCP1, CIDEA, CPT1A, NRF1, TFAM) and enhanced mitochondrial oxidative capacity. **B** Adipose–liver crosstalk in MASLD. Thermogenic adipocytes reduce circulating FFAs by coupling lipolysis with oxidation, thereby limiting hepatic lipid influx. In parallel, endocrine signaling via batokines (FGF21, adiponectin, Nrg4, and irisin) modulates hepatic metabolism through pathways such as AMPK activation and suppression of SREBP-1c -driven lipogenesis. Notably, many of these mechanistic pathways are derived from murine or in vitro models, and their magnitude and consistency in human MASLD remain variable. Thus, browning-related signaling should be interpreted as part of an integrated metabolic network rather than a standalone therapeutic axis. Most molecular interactions illustrated are derived from experimental and preclinical studies; corresponding evidence in human MASLD remains incomplete for several pathways. The figure was created with BioRender.com; a publication license was obtained

In sum, these mechanisms position adipose tissue as a primary upstream regulator of hepatic lipid homeostasis in MASLD. Dysregulation of adipose lipolysis-particularly within visceral depots—drives excessive FFA flux to the liver, while transporter-mediated uptake and intracellular lipid handling amplify hepatic lipid accumulation and lipotoxic stress. In contrast, adipose tissue browning reshapes this axis by enhancing fatty acid oxidation, reducing circulating lipid availability, and indirectly modulating hepatic substrate uptake. Importantly, this adipose–liver interaction is not governed by a single pathway but by the coordinated integration of hormonal regulation, depot-specific lipid release, and transporter activity. While preclinical evidence strongly supports the capacity of browning to mitigate these processes, its overall impact on human MASLD is likely constrained by inter-individual variability in thermogenic capacity and adipose tissue distribution. Thus, adipose lipid flux regulation represents a key mechanistic node within the adipose–liver axis, linking cellular energy metabolism to systemic metabolic outcomes in MASLD. Building on this framework of lipid flux regulation, adipose browning further engages transcriptional and mitochondrial programs that sustain thermogenic function. The molecular signaling pathways and inter-organ crosstalk driving adipose tissue browning are further illustrated in Fig. 2.

#### Browning-associated Thermogenic Gene Program

The acquisition of a thermogenic phenotype in adipocytes is driven by coordinated activation of genes involved in mitochondrial respiration and energy dissipation. A central component of this program is uncoupling protein 1 (UCP1), which disrupts the proton gradient across the inner mitochondrial membrane, allowing energy to be released as heat rather than stored as adenosine triphosphate (ATP) [60, 61]. This thermogenic gene program is primarily induced by β-adrenergic signaling, which activates cyclic adenosine monophosphate–protein kinase A (cAMP–PKA) pathways and downstream transcriptional regulators [62]. Coactivators such as peroxisome proliferator-activated receptor gamma coactivator 1-alpha (PGC-1α) and transcriptional regulators such as PRDM16 coordinate chromatin remodeling and transcriptional activation of thermogenic genes [16].

Functionally, this program enhances mitochondrial density and oxidative capacity, increasing fatty acid utilization and reducing systemic lipid availability. In MASLD, this shift alleviates hepatic lipid accumulation and improves overall metabolic homeostasis.

#### Core Transcriptional Regulators and Mitochondrial–oxidative Machinery

Adipose browning is controlled by a network of transcriptional regulators that integrate environmental and hormonal signals. PR domain containing 16 (PRDM16) promotes brown adipocyte identity [63, 64], while peroxisome proliferator-activated receptor gamma (PPARγ) maintains adipocyte differentiation and lipid handling capacity [65]. Peroxisome proliferator-activated receptor alpha (PPARα) regulates genes involved in fatty acid oxidation [66], linking transcriptional control to metabolic function.

Mitochondrial biogenesis is driven by nuclear respiratory factor 1 (NRF1) and mitochondrial transcription factor A (TFAM), which regulate mitochondrial DNA replication and transcription [67, 68]. This expansion of mitochondrial content supports increased oxidative metabolism required for thermogenesis. Carnitine palmitoyltransferase 1 A (CPT1A) serves as a key regulatory enzyme controlling mitochondrial fatty acid entry [69].

Together, these regulators establish a transcriptional–metabolic framework that supports sustained oxidative flux and thermogenic activity. Disruption of this network contributes to impaired lipid oxidation and metabolic dysfunction in MASLD.

### Liver → Adipose Axis: Hepatic Control of Adipose Function and Browning

In addition to receiving metabolic input, the liver actively regulates adipose tissue function through endocrine and metabolic signaling. Hepatic processes such as de novo lipogenesis and VLDL–triglyceride export influence systemic lipid availability and adipose storage dynamics. In parallel, hepatokines—most notably FGF21—act on adipose tissue to promote thermogenic activation, enhance lipid oxidation, and improve insulin sensitivity. This liver-to-adipose signaling axis represents a compensatory mechanism aimed at restoring metabolic balance under conditions of nutrient excess. However, in chronic metabolic disease, dysregulation of hepatokine signaling and persistent lipid oversupply may impair adipose responsiveness and limit browning capacity. This axis is particularly relevant in the progression from steatosis to MASH, where hepatic signaling begins to influence systemic metabolic adaptation.

#### Batokine-mediated Adipose–liver Endocrine Axis

Batokines influence hepatic metabolism through endocrine pathways. FGF21 is a central mediator that signals through fibroblast growth factor receptor 1 (FGFR1) in complex with β-Klotho, activating downstream pathways such as extracellular signal-regulated kinase (ERK) and AMP-activated protein kinase (AMPK). These pathways enhance hepatic fatty acid oxidation and improve insulin sensitivity [70–72].

Neuregulin 4 (Nrg4) acts via the Erb-B2 receptor tyrosine kinase 4 (ErbB4) to suppress sterol regulatory element-binding protein 1c (SREBP-1c)-driven lipogenesis, reducing hepatic triglyceride synthesis [73]. Irisin, derived from fibronectin type III domain-containing protein 5 (FNDC5), signals through integrin receptors (αVβ5), activating mitogen-activated protein kinase (MAPK) pathways that promote thermogenic gene expression [74]. These endocrine factors provide a mechanistic link between adipose tissue activity and hepatic gene regulation, influencing lipid metabolism, glucose homeostasis, and inflammatory signaling.

#### Endocrine Integration and Feed-forward Adipose–liver Signaling Loops

Adipose-derived signals modulate hepatic metabolism through receptor-mediated pathways that converge on transcriptional regulators of lipid and glucose metabolism. Activation of AMP-activated protein kinase (AMPK), mitogen-activated protein kinase (MAPK), and nuclear receptor pathways influences gene expression programs governing fatty acid oxidation, lipogenesis, and gluconeogenesis.

Reciprocal signaling between adipose tissue and the liver establishes feed-forward loops that sustain metabolic adaptation. For example, hepatic FGF21 enhances adipose browning [75, 76], which in turn increases sensitivity to FGF21 signaling. Similarly, adiponectin activates hepatic AMPK, promoting fatty acid oxidation while improving adipose insulin sensitivity [77, 78].

Disruption of these feedback systems in MASLD leads to impaired metabolic coordination, favoring lipid accumulation and inflammation. Restoration of these loops through adipose browning represents a key therapeutic mechanism. This mechanism lies at the interface of the liver-to-adipose and bidirectional feedback axes, where endocrine factors coordinate systemic metabolic adaptation. It influences multiple stages of MASLD, with effects ranging from modulation of hepatic lipid metabolism in steatosis to attenuation of inflammation in MASH.

### Bidirectional Feedback: Integrated Adipose–liver Crosstalk

The adipose tissue–liver axis operates through dynamic bidirectional feedback loops that integrate metabolic, endocrine, and inflammatory signals. Hepatic factors such as FGF21, bile acids, and microbiota-derived metabolites could promote adipose browning and thermogenic activation. In turn, browned adipose tissue modulates hepatic metabolism through reduced lipid flux and secretion of batokines, including Nrg4 and irisin, which suppress lipogenesis and inflammation. These feedback mechanisms establish an adaptive network that maintains systemic energy homeostasis. However, in MASLD, disruption of this coordination leads to persistent lipid overload, chronic inflammation, and progressive fibrosis. Within this framework, adipose browning contributes to all stages of MASLD but exerts its strongest effects at early and intermediate stages, with more limited impact once advanced inflammation and fibrosis are established.

#### Intracellular Energy-sensing and Metabolic Signaling Pathways Metabolic Signaling Pathways

Cellular energy homeostasis is maintained by signaling networks that integrate nutrient availability with metabolic responses. AMPK is activated by increased adenosine monophosphate to adenosine triphosphate (AMP/ATP) ratios and promotes catabolic processes such as fatty acid oxidation while inhibiting lipogenesis through suppression of acetyl-coenzyme A carboxylase (ACC) and sterol regulatory element-binding protein 1c (SREBP-1c) [79, 80].

The sirtuin 1 (SIRT1)–PGC-1α axis enhances mitochondrial function by promoting transcriptional programs associated with oxidative metabolism [81, 82]. In contrast, the mechanistic target of rapamycin complex 1 (mTORC1) responds to nutrient abundance by stimulating anabolic pathways, including lipid synthesis [83]. Dysregulation of mTORC1 contributes to hepatic steatosis and insulin resistance [84].

Insulin signaling through the IRS–PI3K–AKT cascade integrates hormonal and nutrient signals to regulate glucose and lipid metabolism. Crosstalk among AMPK, SIRT1, and mTOR pathways determines the overall metabolic state, and restoration of this balance is a key effect of adipose browning.

#### Gut Microbiota-derived Signaling Modulation

Gut microbiota-derived metabolites influence host metabolism through receptor-mediated and epigenetic mechanisms. Short-chain fatty acids (SCFAs), including butyrate, acetate, and propionate, activate G protein-coupled receptors such as G protein-coupled receptor 41 (GPR41) and G protein-coupled receptor 43 (GPR43) [85]. These pathways enhance adipose oxidative metabolism and improve insulin sensitivity while also modulating gene expression through inhibition of histone deacetylases [86].

Bile acid signaling represents another critical regulatory axis. Microbial modification of bile acids alters activation of farnesoid X receptor (FXR) and Takeda G protein-coupled receptor 5 (TGR5), which regulate lipid metabolism, glucose homeostasis, and thermogenesis [87–89]. Activation of TGR5 in adipose tissue promotes energy expenditure, while FXR signaling in the liver modulates lipogenesis and bile acid synthesis [90].

Microbial components such as lipopolysaccharide (LPS) activate toll-like receptor 4 (TLR4), triggering inflammatory signaling cascades that impair insulin signaling and promote hepatic lipid accumulation. These pathways collectively link gut-derived signals to adipose and hepatic metabolism, shaping disease progression in MASLD. This pathway represents a key component of bidirectional feedback within the adipose–liver axis, integrating gut-derived signals with host metabolic regulation. Its effects span across disease stages but may be particularly important in modulating inflammation and metabolic flexibility during MASH progression.

Adipose tissue browning influences hepatic metabolism through converging pathways—namely lipid flux, endocrine signaling, energy sensing, mitochondrial adaptation, and immune modulation—which collectively dictate hepatic substrate availability, inflammation, and metabolic flexibility during MASLD progression. Currently, adipose lipolysis, portal FFA flux, and FGF21-mediated signaling boast the strongest evidence linking adipose function to liver metabolism. In contrast, emerging pathways (e.g., batokines, extracellular vesicles) are primarily supported by preclinical models. Importantly, since murine thermogenic responses may not fully mirror human biology, translating these pathways to human MASLD remains a critical focus for future research.

## Recent Advances and Emerging Directions in Adipose Browning and MASLD

### Reappraisal of Human Brown Adipose Tissue and Its Heterogeneity

Recent advances in imaging and metabolic assessment have reshaped our understanding of BAT in humans, revealing substantial heterogeneity in both its distribution and activity. Although BAT was previously considered negligible in adults, positron emission tomography–computed tomography (PET/CT) and emerging imaging modalities now demonstrate that metabolically active BAT is present in specific depots, particularly in the cervical and supraclavicular regions. [7, 91, 92]. However, BAT mass and activity vary markedly across individuals and are strongly influenced by age, environmental exposure, and metabolic status [25, 93, 94]. Importantly, BAT activity is significantly reduced in individuals with obesity, insulin resistance, and MASLD, populations in which thermogenic capacity is most clinically relevant [95–98]. This reduced thermogenic capacity limits the magnitude of achievable increases in energy expenditure and substrate utilization, even when BAT is pharmacologically or physiologically stimulated. While preclinical models consistently show that activation of BAT or induction of browning improves energy expenditure, lipid handling, and hepatic steatosis, the magnitude of these effects in humans appears more modest and variable.

These translational discrepancies are further compounded by differences in adipose depot distribution, sympathetic innervation, and receptor sensitivity between species [24, 99]. Together, these factors highlight that the therapeutic impact of adipose browning in humans is likely constrained and context-dependent, necessitating cautious interpretation of preclinical findings when extrapolating to MASLD pathophysiology and treatment. As such, inter-individual variability in BAT activity represents a critical determinant of therapeutic response and underscores the need for patient stratification and tailored intervention strategies [94, 99].

Another key factor underlying these translational discrepancies is the marked difference in thermogenic capacity between rodent models and adult humans. In rodents, brown adipose tissue represents a relatively large and highly metabolically active organ, and inducible browning of white adipose depots—particularly in subcutaneous regions such as inguinal WAT—is robust and readily sustained under experimental conditions [24, 100]. In contrast, adult humans possess substantially lower BAT mass, and its contribution to whole-body energy expenditure under physiological conditions is comparatively modest [101, 102]. Moreover, thermogenic responsiveness is significantly attenuated in individuals with obesity, insulin resistance, and MASLD, in whom BAT volume and activity are consistently reduced [94, 103].

These quantitative and qualitative differences impose a ceiling on the metabolic impact of browning-based interventions in humans. Even when BAT is pharmacologically activated or stimulated through environmental or behavioral interventions, the magnitude of increase in energy expenditure and substrate utilization remains limited relative to that observed in rodent models. As a result, interventions that produce pronounced improvements in adiposity, insulin sensitivity, and hepatic steatosis in preclinical settings often translate into modest and variable outcomes in clinical studies [104–106]. Taken together, these differences highlight that the efficacy of adipose browning is inherently constrained by baseline thermogenic capacity and disease context in human populations. Thus, while rodent models reflect a system in which thermogenesis is a major determinant of metabolic homeostasis, human physiology appears to rely less on BAT-driven energy dissipation, limiting the standalone therapeutic potential of browning.

### Batokine-mediated Endocrine Communication Along the Adipose—Liver Axis in MASLD

Among the mechanisms linking thermogenic adipose tissue to hepatic metabolism, endocrine communication mediated by batokines has emerged as one of the most extensively investigated pathways. However, despite strong mechanistic evidence from preclinical models, the translational significance of individual batokines in human MASLD remains incompletely defined.

FGF21 is currently the most clinically advanced batokine-linked pathway. In experimental models, FGF21 promotes adipose tissue browning, enhances mitochondrial oxidative metabolism, improves insulin sensitivity, and suppresses hepatic steatosis and inflammation through activation of FGFR1/β-klotho signaling pathways [66–68, 107]. Multiple MASLD and MASH animal studies demonstrate improvements in hepatic lipid accumulation, inflammation, and fibrosis following pharmacologic or genetic augmentation of FGF21 signaling [68, 108]. Translation into humans has been partially successful, as FGF21 analogs such as efruxifermin significantly reduce liver fat and improve metabolic biomarkers in clinical trials [95, 96]. Nevertheless, histological responses are variable, and therapeutic effects likely reflect systemic metabolic reprogramming rather than browning-dependent mechanisms alone [95–97].

Neuregulin 4 (Nrg4) represents another important mediator of adipose–liver communication. Nrg4 is highly expressed in thermogenic adipose tissue and suppresses hepatic lipogenesis through inhibition of SREBP1c–dependent pathways [69, 109]. Experimental studies demonstrate protection against steatosis, inflammation, and metabolic dysfunction following Nrg4 activation [109]. However, human evidence remains inconsistent. Although reduced circulating Nrg4 levels have been reported in some studies of metabolic disease, meta-analyses have identified substantial heterogeneity and conflicting associations with fatty liver disease [110]. These discrepancies may reflect differences in study populations, disease stage, adiposity, and thermogenic capacity.

Irisin provides an additional mechanism through which exercise, skeletal muscle, and adipose tissue interact to influence metabolic homeostasis. Preclinical studies show that irisin promotes browning, enhances thermogenic gene expression, improves mitochondrial function, and attenuates hepatic steatosis and inflammatory signaling [70, 111, 112]. However, human studies have yielded variable findings, partly because circulating irisin concentrations are influenced by age, physical activity, body composition, and methodological differences in measurement. Consequently, its physiological importance and therapeutic potential remain subjects of ongoing debate.

Importantly, the translational effects of batokine signaling are likely attenuated in human MASLD by several biological factors. Individuals with obesity, insulin resistance, and advanced metabolic disease commonly exhibit reduced BAT activity, diminished browning capacity, chronic low-grade inflammation, and impaired endocrine responsiveness, all of which may blunt downstream signaling effects [89, 91–94]. Furthermore, receptor expression, signaling efficiency, and tissue sensitivity may differ substantially between rodents and humans. As a result, endocrine pathways that exert large effects in highly controlled animal models frequently produce more modest and variable outcomes in clinical studies.

Taken together, available evidence supports batokines as important mediators of adipose–liver crosstalk and promising therapeutic targets. However, current human evidence remain insufficient to establish the magnitude of their contribution to MASLD progression or treatment. Accordingly, findings from preclinical models should be interpreted cautiously, and future studies should seek to identify patient populations most likely to benefit from modulation of batokine signaling.

### Translational Progress: FGF21-based Therapies and Adipose–Liver Signaling

Among emerging therapeutic strategies, FGF21 analogs represent one of the most advanced efforts to translate adipose–liver crosstalk into clinical intervention. FGF21 mimetics such as efruxifermin have demonstrated promising effects in clinical trials, including reductions in liver fat, improvements in metabolic biomarkers, and signals of fibrosis regression in patients with metabolic dysfunction–associated steatohepatitis (MASH) [113, 114].

Longer-term studies suggest that sustained FGF21 signaling may contribute to histologic improvements in fibrosis, although early endpoints are not consistently met, underscoring the complexity of therapeutic translation. Notably, FGF21 exerts effects across multiple tissues, including adipose depots, where it enhances thermogenic activation and metabolic flexibility, supporting the concept that systemic metabolic reprogramming—not liver-specific targeting alone—is required for therapeutic efficacy [115].

### Limitations of β3-Adrenergic Agonists and the “Browning Paradox”

Parallel to advances in endocrine therapies, pharmacological activation of BAT using β3-adrenergic receptor agonists such as mirabegron has provided critical insights into the limitations of browning-based interventions. Human studies confirm that mirabegron increases BAT activity, resting energy expenditure, and lipolysis. However, meta-analyses and clinical studies demonstrate minimal or inconsistent effects on key metabolic endpoints such as BAT volume, glycemic control, and sustained weight loss [116]. Additionally, off-target cardiovascular effects, including increased heart rate and blood pressure, pose translational challenges. Collectively, these findings highlight a critical “browning paradox” representing that the activation of thermogenesis does not necessarily translate into meaningful disease modification in humans. This paradox underscores the need to reposition adipose browning within a broader therapeutic framework that accounts for its limited standalone efficacy but potential synergistic value [99, 100].

Several mechanistic factors likely underlie this browning paradox. First, although activation of BAT increases resting energy expenditure in humans, the absolute magnitude of this increase is relatively small compared with total daily energy expenditure, thereby limiting its impact on body weight and systemic metabolism [100, 101]. Second, compensatory physiological responses may attenuate net energy balance, including increased appetite or adaptive reductions in spontaneous physical activity, which can offset the modest thermogenic gains achieved through BAT activation [100]. Third, pharmacologic stimulation of β3-adrenergic pathways is constrained by off-target cardiovascular effects, such as increased heart rate and blood pressure, which limit the use of higher or sustained dosing that might otherwise produce greater metabolic benefits [102, 106].

It is noteworthy that the responsiveness of adipose tissue to browning stimuli is not uniform across populations. In individuals with long-standing obesity, insulin resistance, and advanced MASLD, adipose tissue exhibits reduced plasticity, impaired mitochondrial function, and diminished thermogenic capacity [24, 94, 95, 102]. These factors further attenuate the effectiveness of browning-based interventions in precisely those populations where therapeutic benefit is most needed. Together, these considerations indicate that the metabolic impact of browning in humans is both quantitatively limited and highly context-dependent.

Taken together, these findings support a reframing of adipose browning within the therapeutic landscape of MASLD. Rather than functioning as a standalone intervention, browning should be viewed as an upstream metabolic modulator that enhances lipid utilization and systemic metabolic efficiency. Its clinical value is therefore likely to be maximized when integrated with complementary strategies, including weight loss interventions, caloric restriction, incretin-based therapies, and agents targeting hepatic metabolism and inflammation. This combinatorial approach aligns with evidence from both preclinical and clinical studies demonstrating that browning exerts synergistic effects when coupled with broader metabolic interventions.

### Expansion of the Adipose–liver Axis: Gut Microbiota and Metabolic Signaling

A major conceptual advance is the integration of the gut microbiome into the adipose–liver axis. Gut microbiota–derived metabolites, particularly short-chain fatty acids (SCFAs), have emerged as key regulators of host metabolism [117, 118] SCFAs such as acetate, propionate, and butyrate act as signaling molecules through G protein–coupled receptors including GPR41 and GPR43, modulating energy homeostasis, inflammation, and lipid metabolism [119]. These pathways influence adipose tissue browning by enhancing mitochondrial function, promoting thermogenic gene expression, and integrating nutrient availability with systemic metabolic responses. Furthermore, SCFAs participate in gut–brain signaling, linking microbial activity to central regulation of energy balance. Despite robust preclinical evidence, translation to human metabolic disease remains limited, and the extent to which microbiome-driven browning contributes to MASLD pathogenesis requires further investigation.

### Recognition of Inter-individual Variability in Browning Capacity

Another major advance is the recognition that thermogenic capacity is highly heterogeneous across individuals. BAT activity declines with aging and is markedly reduced in obesity, contributing to diminished metabolic responsiveness [98, 120]. This variability likely explains the discordance between preclinical success and clinical outcomes. Individuals with detectable BAT (“BAT-positive phenotype”) exhibit more favorable metabolic profiles, yet the proportion of such individuals decreases with metabolic disease progression. These observations emphasize that future therapeutic strategies must account for patient stratification, rather than assuming uniform responsiveness to browning-based interventions.

An additional challenge in interpreting human BAT studies is distinguishing causality from association. Individuals with detectable BAT frequently exhibit a healthier cardiometabolic phenotype, including lower adiposity, improved insulin sensitivity, and more preferred lipid profiles [120–122]. However, whether BAT directly drives these metabolic advantages or is preferentially retained in metabolically healthy individuals remains uncertain. This distinction is particularly important in MASLD, where adipose tissue dysfunction, insulin resistance, and systemic inflammation may simultaneously suppress BAT activity and promote disease progression, creating the appearance of association without necessarily proving causation [96].

### Barriers to Clinical Translation of Adipose Browning

Despite the strong mechanistic rationale supporting adipose tissue browning as a therapeutic target, several important barriers constrain its clinical translation in MASLD. One major challenge is the limited sustainability of browning activation. Many currently available stimuli -including cold exposure, pharmacologic β3‑adrenergic activation, and metabolic signals—induce only transient thermogenic responses, with effects often diminishing once the intervention is withdrawn. Sustained activation of thermogenic pathways in a safe and controlled manner remains difficult to achieve in human settings [123, 124]. In addition, commonly studied approaches to induce browning present practical and safety-related limitations. Cold exposure, while effective in activating BAT, is logistically challenging and unlikely to be a sustainable long-term intervention, with human studies demonstrating limited impact on weight-related outcomes and feasibility concerns [125, 126]. Similarly, β3‑adrenergic agonists such as mirabegron increase BAT activity and energy expenditure in humans; however, their metabolic effects are variable and may attenuate over time, and their clinical use is constrained by off-target cardiovascular effects, including increases in heart rate and blood pressure [104, 123, 127]. Other metabolic therapies, including endocrine mediators such as FGF21 analogs and exercise-induced signaling pathways, show variable efficacy, and their metabolic benefits are not always directly attributable to browning or may not consistently produce durable thermogenic responses across populations [128, 129].

Furthermore, issues of clinical feasibility and acceptability remain significant obstacles. Inter-individual variability in BAT mass and responsiveness contributes to heterogeneous treatment outcomes, particularly in populations with obesity or advanced MASLD, where thermogenic capacity is already reduced [93, 126]. These constraints highlight that adipose browning is unlikely to provide uniform therapeutic benefit across all patients. Taken together, these limitations underscore the need to view adipose browning not as a standalone intervention, but as part of a broader, integrated therapeutic strategy.

### Shift Toward Combination Therapy Strategies

Emerging evidence suggests that adipose browning is most effective when integrated with complementary metabolic interventions [19, 100, 130]. For example, in preclinical models of steatohepatitis, β3-adrenergic stimulation alone improves metabolic parameters but fails to reverse hepatic inflammation; however, when combined with caloric restriction, it produces significant improvements in steatosis, inflammation, and overall disease activity [131]. This supports a growing consensus that browning acts synergistically with weight loss, dietary interventions, and pharmacologic therapies rather than functioning independently. More broadly, combination approaches incorporating incretin-based therapies, mitochondrial modulators, and anti-inflammatory agents may enhance the efficacy of browning by targeting multiple nodes of metabolic dysfunction simultaneously.

### Toward a Systems-level Model of MASLD

Taken together, these advances converge on a revised conceptual framework in which adipose browning operates within a broader network of inter-organ metabolic communication. MASLD is increasingly understood as a systemic disorder involving coordinated dysregulation across adipose tissue, liver, gut, and central nervous system [11]. In this context, adipose browning contributes to metabolic homeostasis through three interconnected mechanisms: (i) redistribution of lipid flux away from the liver; (ii) endocrine signaling via batokines and adipokines and (iii) integration into gut–brain–adipose feedback networks. However, recent data also suggest that browning alone is insufficient to reverse advanced disease, highlighting the need for integrated strategies that leverage adipose plasticity within a multi-target therapeutic framework [132].

Building on this system-level framework, it is critical to identify which molecular pathways and biomarkers are most relevant for clinical translation. While numerous molecular pathways involved in adipose browning have been identified, not all demonstrate consistent translational relevance in humans. Among these, key regulatory axes such as AMPK–PGC-1α signaling, PRDM16-mediated transcriptional control, and the FGF21–FGFR1/β-klotho endocrine pathway appear to be the most robustly supported across both preclinical and emerging clinical studies. These pathways integrate mitochondrial biogenesis, substrate utilization, and systemic metabolic signaling, making them promising targets for therapeutic modulation in MASLD.

In parallel, the identification of reliable biomarkers remains critical for patient stratification and treatment monitoring. Current evidence supports the use of functional markers such as UCP1 expression and BAT activity assessed by PET/CT imaging as direct indicators of thermogenic capacity. In addition, circulating factors including FGF21, adiponectin, and Nrg4 show potential as minimally invasive biomarkers reflecting adipose–liver crosstalk. However, heterogeneity in BAT distribution and responsiveness suggests that composite biomarker approaches integrating imaging, circulating factors, and transcriptional profiling may be necessary to identify patients most likely to benefit from browning-based interventions.

## Functional Outcomes of Adipose Browning with Translational Evidence

**Fig. 3:**
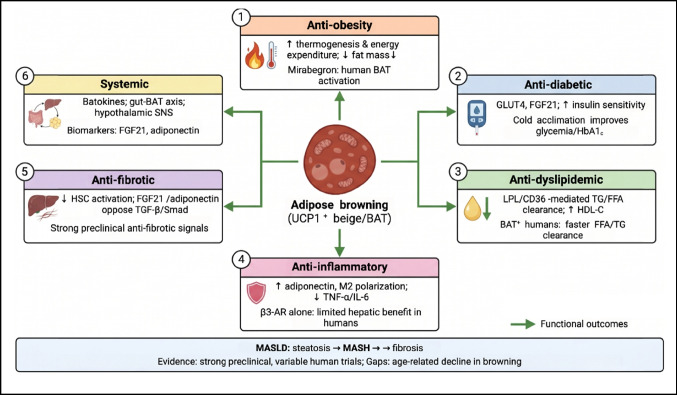
Functional and translational outcomes of adipose tissue browning in MASLD

Building upon the mechanistic pathways discussed above, the following section focuses on the functional consequences of adipose tissue browning and highlights how these interconnected mechanisms translate into hepatic and systemic metabolic outcomes. How does adipose tissue browning translate molecular mechanisms into integrated systemic and hepatic outcomes relevant to MASLD is summarized in Fig. 3. Thermogenic activation enhances energy expenditure and reduces adiposity while improving insulin sensitivity through mediators such as GLUT4 and FGF21. Concurrently, lipid handling is optimized via increased triglyceride and free fatty acid clearance (LPL, CD36), limiting hepatic substrate overload. Browning also attenuates inflammatory signaling (reduced TNF‑α and IL‑6; increased adiponectin) and suppresses fibrogenesis by inhibiting hepatic stellate cell activation and TGF‑β–driven pathways. These effects are further coordinated by endocrine (batokines) and neuro-metabolic signaling, collectively modulating disease progression from steatosis to fibrosis.

Despite growing evidence linking BAT activity and adipose tissue browning to improved metabolic health, important uncertainties regarding causality remain. Many human studies rely on cross-sectional PET/CT imaging analyses or observational cohort designs showing that individuals with detectable BAT exhibit lower body mass index, improved insulin sensitivity, and more favorable lipid profiles [120–122]. However, these associations do not establish whether BAT directly mediates these metabolic benefits or whether preserved BAT activity simply reflects a healthier underlying metabolic phenotype [93, 99]. Furthermore, most interventional studies examining BAT activation are relatively short in duration and primarily evaluate surrogate metabolic endpoints rather than long-term hepatic outcomes. Consequently, current human evidence should be interpreted as supportive but not definitive proof of a causal role for adipose browning in preventing or reversing MASLD progression.

Schematic overview of the systemic and hepatic consequences of adipose tissue browning, integrating molecular mechanisms into physiologically relevant outcomes in MASLD. Thermogenically active beige and brown adipocytes (UCP1-positive) exert multi-dimensional metabolic effects across several domains: (1) Energy balance and adiposity – increased thermogenesis elevates energy expenditure and reduces fat mass; (2) Glucose homeostasis – enhanced glucose uptake (e.g., via GLUT4 and FGF21 signaling) improves insulin sensitivity; (3) Lipid metabolism – Increased lipoprotein lipase (LPL) activity and fatty acid transport facilitate clearance of triglycerides and circulating FFAs, limiting hepatic lipid influx; (4) Inflammation – browning promotes an anti-inflammatory milieu characterized by increased adiponectin and reduced pro-inflammatory cytokines (e.g., TNF-α and IL-6); and (5) Fibrogenesis – indirect suppression of hepatic stellate cell activation and TGF‑β/Smad signaling pathways attenuates fibrotic progression. These effects are further coordinated through endocrine and neuro-metabolic pathways, including batokine signaling, gut–adipose interactions, and sympathetic nervous system input, forming an integrated multi-organ regulatory network. However, while these outcomes are consistently demonstrated in preclinical models, translation to human MASLD remains variable and often modest, with effect size influenced by factors such as BAT activity, aging, metabolic status, and the durability of browning induction. Accordingly, adipose browning is best interpreted as an upstream metabolic modulator that complements, rather than replaces, established therapeutic strategies. The figure was created with BioRender.com; a publication license was obtained.

### Anti-obesity and Energy Expenditure Effects

Beige and brown adipocytes are defined, almost at a structural level, by the dense presence of uncoupling protein 1 (UCP1) along the inner mitochondrial membrane. What this arrangement permits is a controlled proton leak, effectively decoupling oxidative phosphorylation from ATP production and redirecting that energy into heat release [133]. The consequence is not subtle. Under cold exposure or targeted β3-adrenergic stimulation, mitochondrial oxygen consumption rises sharply, and basal metabolic rate follows [134, 135].

In murine systems, attempts to expand the beige adipocyte pool, often grouped under the term “browning,” consistently blunt weight gain. The data are direct. Transplanting active brown adipose tissue into obese mice reduces fat mass, improves insulin responsiveness, and limits hepatic lipid accumulation [107, 136]. Comparable outcomes appear when browning is driven pharmacologically or through genetic manipulation, particularly under high-fat diet conditions where excess lipid would otherwise accumulate [137, 138].

The mechanism, at least in part, seems to rest on substrate partitioning. Thermogenic adipocytes draw in circulating free fatty acids and triglycerides, using them as fuel rather than allowing deposition in non-adipose tissues such as the liver [133, 139]. This becomes especially relevant in metabolic dysfunction-associated steatotic liver disease (MASLD), where white adipose tissue browning shifts whole-body energy balance and reduces hepatocellular lipid load [135, 140].

Human physiology complicates the picture slightly. Brown adipose tissue mass is lower than in rodents, which initially raised doubts about its relevance. Yet imaging studies using positron emission tomography suggest otherwise. Individuals with detectable BAT tend to present with lower body mass indices and more favorable glycemic control than those without measurable activity [120, 121]. Acute cold exposure rarely produces dramatic weight loss. Sustained activation is another matter. Agents such as mirabegron, administered over roughly twelve weeks, have been associated with reductions in visceral fat and measurable metabolic improvement [106, 141]. Circulating factors, including FGF21 and adiponectin, track closely with this phenotype and show inverse relationships with obesity-linked complications in clinical cohorts [142, 143].

### Anti-diabetic and Insulin-sensitizing Effects

From a glucose regulation standpoint, beige adipocytes function less as passive storage units and more as active metabolic regulators. Their elevated energy demand necessitates increased glucose uptake, largely mediated by the upregulation of glucose transporter 4 (GLUT4) under cold exposure or β3-adrenergic signaling [136, 144]. This is only part of the story. Activated thermogenic fat also secretes signaling molecules, often grouped as batokines. Among these, FGF21 stands out for its capacity to enhance basal glucose uptake through GLUT1 expression [145, 146].

Cellular energy sensing pathways intersect here. AMP-activated protein kinase (AMPK), activated during browning, promotes mitochondrial biogenesis and shifts substrate use toward fatty acid oxidation [133, 147]. The downstream effect is a reduction in lipotoxic intermediates such as diacylglycerols and ceramides, both of which interfere with insulin signaling in liver and skeletal muscle [148]. Less interference, better signaling.

Evidence across models aligns, though not perfectly. UCP1-deficient mice, when placed under metabolic stress, develop insulin resistance more readily, which reinforces the functional role of thermogenesis [149]. Human data echo this trend. Chondronikola et al. reported that individuals with detectable BAT exhibit higher whole-body glucose disposal and improved insulin sensitivity following mild cold acclimation compared to matched controls [121, 122]. Pharmacological studies using mirabegron report similar improvements, including reductions in HbA1c, with effects correlating to the presence of beige adipocyte markers in subcutaneous white adipose tissue [106]. Even conventional therapies may intersect with this pathway. DPP-4 inhibitors such as sitagliptin have been suggested to induce subtle browning effects, though the extent of this contribution remains under discussion [141, 150].

### Anti-dyslipidemic Effects

The influence of thermogenic adipose tissue extends into lipid handling, particularly in how circulating lipoproteins are processed. Beige and brown adipocytes express elevated levels of lipoprotein lipase (LPL) and the fatty acid transporter CD36, which together facilitate the breakdown of triglyceride-rich lipoproteins, including chylomicrons and very-low-density lipoproteins [136, 151]. The remnants generated through this process are then cleared hepatically via ApoE-dependent LDL receptor pathways [151, 152].

What follows is a shift in lipid profile. Circulating triglyceride levels decline, while high-density lipoprotein cholesterol becomes relatively enriched, producing a less atherogenic state [153, 154]. In mouse models, the effect size is difficult to ignore, with fasting triglycerides reduced by as much as 30% to 60% even under lipid-rich dietary conditions [155].

Human observations add some nuance. Overweight women with higher BAT volume display faster clearance rates for both VLDL-triglycerides and free fatty acids compared to those with lower BAT activity [156]. Cohort-level analyses extend this association, linking BAT positivity with higher HDL-C, improved total-to-HDL ratios, and reduced incidence of major cardiovascular events [120, 157]. Mechanistically, the diversion of free fatty acids toward thermogenic oxidation limits their availability for hepatic triglyceride synthesis. This constraint on substrate supply appears to slow, if not partially interrupt, the progression of steatosis and associated inflammatory changes in the liver [105, 135].

### Anti-inflammatory Effects

Browning of adipose tissue shifts the cytokine milieu toward an anti-inflammatory state. Thermogenic adipocytes together with their resident macrophages have been shown to suppress pro-inflammatory cytokines and up-regulate adiponectin alongside other anti-inflammatory compounds. In fact, treatment with the FXR/TGR5 agonist INT-767 in obese animal models induced WAT browning and reverted the increased pro-inflammatory genes to normal levels in liver and adipose tissue [158]. β3-adrenergic stimulation of BAT in mice would also mitigate systemic inflammation when paired with weight loss [159]. It is important to note, however, that BAT activation alone had little effect on hepatic inflammation. To add, adiponectin, secreted mainly by adipocytes including brown and beige fat, displays potent anti-inflammatory and hepatoprotective capacity [159]. Adiponectin is well-known to show insulin-sensitizing and anti-inflammatory roles in organs including the liver. This blocks TNF-α–induced chemokine expression and macrophage infiltration via the APPL2–mTORC1 pathway [159, 160]. Also, as seen in adiponectin-deficient models, replenishment of this compound reverses fatty liver, restores mitochondrial respiratory-chain activity, and prevents lipid peroxidation together with inflammatory injury [161]. Evidence would also now suggest that adipose browning indirectly lowers hepatic TNF-α and IL-6 effects by promoting adiponectin and M2-like macrophage polarization [162]. Such effects observed include improvement of insulin signaling and reducing oxidative stress in hepatocytes [162]. Batokine signals, such as FGF21, also have anti-inflammatory actions [163, 164]. These mechanistic insights, in totality, suggest that adipose browning tempers adipose-driven inflammation and oxidative stress, thereby protecting the liver from inflammation-driven injury.

Increased browning consistently attenuated inflammatory liver injury in animal nonalcoholic steatohepatitis models. In a study looking into the metabolic effects of nonshivering thermogenesis, a β3-agonist that reactivated BAT reduced steatosis and body weight in obese diabetic foz/foz mice with established MASH but did not by itself reverse liver inflammation [131]. However, when BAT stimulation was combined with caloric restriction, the model showed markedly lower hepatic inflammation and injury [131]. These findings then demonstrate that BAT activation would have synergistic effects with metabolic stress relief to curb hepatic inflammatory signals thereby slowing MASH progression. Preclinical studies also investigated direct BAT transplantation or browning-inducing therapies. These studies reported lowered hepatic expression of TNF-α, IL-6, MCP-1 and other inflammatory genes with notable correlation with reduced stellate activation [108, 165]. Small-scale studies involving human participants also hint at anti-inflammatory benefits, which would include lower levels of circulating TNF-α and IL-6 and higher adiponectin [166, 167]. Another interesting note is that cold exposure studies in healthy volunteers concluded that acute BAT activation leads to release of IL-6 that subsequently improves systemic glucose tolerance without pro-inflammatory implications [168, 169]. With all of these, evidence from rodent models and random clinical trials would support that adipose browning can dampen chronic low-grade inflammation, attenuate hepatocyte injury and slow the transition from steatosis to MASH.

### Anti-fibrotic and Tissue-protective Effects

Adipose browning secondarily reduces fibrogenic signaling in the liver by mitigating lipotoxicity and inflammation as mentioned in earlier sections. Browning decreases the hepatic substrates and cytokines that drive stellate cell activation [170]. This may also release endocrine factors that directly inhibit fibrosis [170]. Beige adipocyte activity, in principle, lowers circulating free fatty acids and inflammatory mediators, reducing TGF-β production in the liver [109]. In addition, at the level of the cell, adiponectin [110] and BAT-derived FGF21 [111] are noted to antagonize fibrogenic pathways. For instance, in an in vitro study, adiponectin signaling in the liver suppresses TGF-β/Smad and NF-κB pathways, thereby showing inhibition of hepatic stellate cell activation [171]. Likewise, FGF21, which is secreted by adipose during browning, notably attenuates fibrosis [111]. In murine models of metabolic liver injury, intervention of FGF21 curbed hepatic fibrogenesis and was then associated with a decrease in α-SMA expression and collagen I deposition [172]. To explain such mechanism, FGF21 downregulates TGF-β expression, Smad2/3 phosphorylation and NF-κB signaling in stellate cells, thereby preventing their activation [111, 172].

Browning therapy, at least in controlled laboratory conditions, has shown anti-fibrotic benefits in MASH models. In murine models, again, with diet-induced MASH, browning activation reduced markers of fibrosis [173]. Specifically, the dual FXR/TGR5 agonist INT-767, which drives adipose browning and BAT differentiation, induced a significant decrease of said markers [173]. Browning also indirectly decreases hepatic TGF-β signaling by lowering steatosis and inflammation. As mentioned previously, reduced hepatocellular injury produces less reactive oxygen species and TGF-β, blunting fibrogenesis [171]. In animal studies of advanced disease, treatments that increase BAT thermogenesis or BAT-derived factor administration have led to smaller fibrosis areas and lower hydroxyproline content. Slowed progression of existing fibrosis and improved liver appearance were also noted [174].

### Endocrine and Systemic Effects

Adipose browning also impacts the liver through secreted batokines and through neuroendocrine pathways that are independent of its effects on lipid flux. Activated brown and beige fat release a plethora of endocrine factors with systemic actions. FGF21, for example, while produced by the liver under fasting conditions, is also expressed in thermogenic adipocytes [111]. BAT-derived FGF21 acts on distant tissues to improve insulin sensitivity [121, 122, 175] and may exert direct hepatoprotective effects [111]. Aside from this, neuregulin 4 (Nrg4), an adipokine enriched in brown or beige adipose, binds to ErbB3 receptors on hepatocytes [176]. As seen in murine models, Nrg4 signaling could suppress hepatic lipogenesis and, subsequently, limit MASH progression [176]. It is notable however that human studies have shown mixed results regarding Nrg4 levels in fatty liver [177], suggesting that more research is needed. Irisin, a myokine induced by exercise that also increases with WAT browning, has been noted to protect hepatocytes. In vitro irisin treatment of steatotic hepatocytes inhibited lipogenic enzymes and suppressed oxidative stress and inflammatory signaling [178], proposing alternative anti-inflammatory and anti-lipogenesis mechanisms.

BAT has also been observed to engage in central neuro-metabolic circuits. Sympathetic activation of BAT, as a response to cold or diet, can reset hypothalamic regulators of energy balance and glucose homeostasis [179], indirectly benefiting the liver. Interestingly, there is emerging evidence linking the gut microbiome to adipose browning. certain microbiota-derived metabolites (such as butyrate) promote beige fat induction, potentially creating a liver–gut–BAT axis of metabolism [180]. At the population level, imaging studies show that individuals with higher active BAT volume tend to have better systemic metabolic profiles. Such examples are lower insulin resistance [175] and dyslipidemia [122, 156] that are likely to translate to reduced liver injury. Circulating batokines and markers of BAT activity thus serve as biomarkers of this inter-organ crosstalk. Higher BAT activity correlates with higher circulating adiponectin and FGF21 and lower liver fat content. The “browning paradox” further highlights that increases in thermogenic activity do not necessarily translate into meaningful clinical outcomes when considered in isolation. Accordingly, adipose browning is best positioned as a complementary strategy that supports, rather than replaces, established metabolic therapies in MASLD.

While adipose browning consistently improves metabolic expenditures and hepatic lipid handling in animal models, human studies show more modest, heterogeneous outcomes due to species-specific variations [24, 99, 100]. Nonetheless, adipose browning functions as a critical upstream regulator that enhances systemic glucose homeostasis, lipid handling, and inter-organ communication, thereby attenuating MASLD pathogenesis.

## Browning of White Adipose Tissue in the Era of Precision Medicine

Identifying potential interventions to address MASLD is important to address the needs of the population-at-risk, moreso, in special populations whose physiological response to the pathways leading to the browning of WAT are reduced such as the elderly population [181]. The metabolic benefits of WAT browning expand the number of strategies to address the growing problem with MASLD. Various metabolic markers and receptors that have previously linked with increased expression of brown adipose tissue, such as PPAR and FGF21, are potential targets for intervention amidst the era of precision medicine. Moreover, the role of lifestyle interventions, physical activities and dietary style, were also previously studied. We describe the current practices, emerging approaches, and the pertinent challenges in translating these findings into actual MASLD interventions.

### Pharmacologic Targets and Emerging Approaches

PPAR are receptor proteins regulating the expression of genes significant in lipid metabolism and adipocyte differentiation. This group of proteins is composed of PPARα, PPARγ, and PPARβ/δ [182]. Animal models have shown reductions in inflammation, stimulation of thermogenesis, and induction of browning of white adipose tissue when treated with fenofibrate [183], suggesting their potential role in reducing the incidence of MASLD. While PPAR-β/δ agonists affect insulin resistance and adiposity index, PPARα agonists provide more promising results as a potential pharmacologic target. PPARα agonists induced enhanced UCP1 gene expression, increased irisin levels, and improved insulin resistance [184].

The important role of FGF21, a protein primarily produced in the liver, in the browning of WAT has previously been described [185, 186]. In animal studies, FGF21 is shown to be a pharmacologic target to induce a thermogenic response in BAT and WAT by increasing the expression of thermogenic genes, UCP1 and CIDEA. Moreover, it increases the present PGC-1a protein which plays a role in thermogenesis and oxidative metabolism [145].

More recent studies have begun looking to messenger RNAs and their potential role in the browning of adipose tissue to address MASLD. Some specific proteins or epigenetic changes of interest are N6-methyladenine (m6A) and microRNAs.

N6-methyladenine (m6A) is a prevalent epigenetic modification in mRNA regulated by writers, readers, and erasers. It affects brown adipose tissue and the associated metabolic processes through changes in the gene [187]. Animal studies have shown that m6A-facilitated HIF1A expression affects thermogenesis and the browning of white adipose tissue through a cascade of processes beginning with the deletion of FTO, a gene associated with the regulation of fatty acid mobilization in adipocytes [188]. By this mechanism, there is an eventual increase in PGC-1α and UCP1 expression [189], both of which are important to the browning process of white adipose tissue as previously described.

In addition to m6A-mediated regulation, post-transcriptional mechanisms—particularly those involving microRNAs—have emerged as critical modulators of adipose tissue browning and metabolic regulation. MicroRNAs are non-coding RNA molecules that regulate gene expression and play important roles in adipocyte differentiation, lipid metabolism, and mitochondrial function, thereby influencing thermogenic capacity and systemic metabolic homeostasis [112, 190]. Over recent years, multiple microRNAs have been identified as key regulators of lipid metabolism and hepatic steatosis. Among these, microRNA-22 (miR-22) has been shown to regulate metabolism, inflammation, and cellular differentiation [191]. studies demonstrate that loss-of-function of miR-22 is protective against obesity and hepatic steatosis, whereas its overexpression increases susceptibility to metabolic dysfunction, even under controlled dietary conditions [192]. Similarly, inhibition of miR-143 promotes thermogenesis in brown adipose tissue (BAT) while suppressing white adipose tissue (WAT) adipogenesis, leading to improved metabolic outcomes [73]. The miR-503–BMPR1a axis also plays a critical role in adipocyte differentiation and browning through modulation of insulin receptor substrate-1 (IRS-1) and PI3K/AKT signaling pathways [193]. In addition, other microRNAs, including miR-33, miR-155, and miR-126b-5p, have been implicated in regulating lipid metabolism and adipose tissue function [194].

Importantly, unlike acute stimuli such as cold exposure or adrenergic activation, microRNA-mediated regulation can induce more durable changes in gene expression networks by coordinately modulating entire transcriptional programs involved in thermogenesis and adipocyte remodeling. This system-level regulatory property suggests a potential advantage in sustaining thermogenic activity over time. However, the translational application of microRNA-based therapies remains at an early stage, and further studies are required to evaluate their safety, specificity, and long-term efficacy in human metabolic disease [112, 195].

### Lifestyle-related Interventions

Physical activity has long been established to improve overall cardiovascular and metabolic health, directly affecting the proposed pathogenesis of MASLD: insulin resistance, lipotoxicity, and chronic inflammation [196]. In the context of browning of WAT, sustained physical activity increases the expression of PGC-1α in the skeletal muscles which, through a cascade, eventually releases the myokine Irisin into the bloodstream [197]. Subsequently, Irisin binds onto WAT receptors promoting UCP1 expression to enhance thermogenesis and energy expenditure through conversion of white adipose tissue into beige or brown adipose tissue [196, 198]. Apart from this, exercise was also previously found to induce expression of FGF21, activating the pathways towards induction of thermogenic response as previously described [83]. The duration and type of exercise that would lead to the desired effects is still being explored given the limited studies. In an animal study, high-intensity interval training led to the beneficial browning of WAT [199]. Moreover, the combined aerobic and resistance training in human studies was previously found to promote the thermogenic activity of adipose tissue [200]. In contrast, human studies using moderate and vigorous endurance training alone within 24 weeks did not lead to an increase in BAT volume among young adults [201], like previous findings using a 6-week resistance training [202].

Dietary modification is also an intervention in discussing metabolic conditions. Numerous studies have explored the mechanisms by which different diets affect the browning of adipose tissue. Apart from the resultant increase in free fatty acids in the bloodstream from increased caloric intake, diet-induced MASLD also leads to a defect in the inter-organ metabolic crosstalk impairing the browning of WAT [97]. Combined with HIIT, caloric restriction promotes the browning of WAT and the reduction of visceral fat in animal models more than HIIT alone. This is proposed to be secondary to the activation of the PPARγ/PGC-1α/UCP1 pathway leading to the eventual fat loss in obese rats [203]. Similarly, doing high-intensity interval exercise in the fasted state increases the fat browning-related adipomyokines which was seen to be through the AMPK-SirT1-PGC1α pathway and increased expression of UCP1 in animal models [204]. Intermittent fasting, with its multiple phenotypes, is one of the strategies by which dietary modification is proposed to affect the browning of WAT [205]. Intermittent fasting (IF) was previously found to increase the volume of beige adipose tissue in WAT and improve the proposed mechanisms in the pathogenesis of MASLD through its effects on the gut microbiota [206, 207]. Additionally, another pathway by which IF is proposed to induce browning of WAT is through the GP130/IL23R-p38 Cascade [135].

The effects of cold exposure remain important in the context of browning of WAT. In animal models, those subjected to thermoneutral housing developed increased hepatic fibrosis, as compared to those exposed to lower temperatures where there is expected higher basal metabolic rates and energy expenditure [97]. Cryolipolysis, the process by which localized fat is reduced by a cooling technique, is another intervention being explored with the most recent available study showing promising results [208].

## Conclusion and Future Perspectives

The current body of evidence positions adipose tissue browning as a central modifier of metabolic homeostasis and a compelling upstream target in MASLD. Mechanistically, browning reprograms white adipose tissue into a thermogenically active phenotype that enhances mitochondrial β-oxidation, increases energy expenditure, and reduces circulating free fatty acids, thereby limiting hepatic lipid accumulation. Beyond substrate redistribution, browning exerts systemic effects through endocrine signaling, with batokines such as FGF21, irisin, and Nrg4 modulating hepatic lipid metabolism, insulin sensitivity, and inflammatory pathways. These interconnected processes highlight a tightly regulated adipose–liver axis that influences disease progression across the MASLD spectrum. Preclinical models consistently demonstrate improvements in steatosis, inflammation, and fibrosis; while emerging human studies suggest favorable associations with metabolic health. However, variability in brown adipose tissue activity, limited durability of induced browning, and heterogeneity in human responses underscore the complexity of translating these findings into clinical benefit.

Despite this strong mechanistic rationale, several key factors limit the clinical translation of adipose browning in human MASLD. In adult humans, BAT mass and thermogenic capacity are relatively low, highly variable, and further diminished in obesity and advanced metabolic disease. These features substantially restrict the maximal metabolic impact that can be achieved through browning-based interventions. Moreover, current approaches to stimulate browning—such as cold exposure, β3-adrenergic agonists, and metabolic signaling molecules—often produce modest or short-lived effects and may be associated with limited tolerability or off-target consequences. Consequently, while adipose browning can favorably influence lipid flux, insulin sensitivity, and inflammatory pathways, its overall contribution to clinically meaningful improvements in MASLD is likely to be incremental rather than transformative when applied in isolation. Collectively, adipose browning is best positioned as an upstream metabolic modulator that may enhance the efficacy of broader therapeutic strategies, including lifestyle interventions and pharmacologic treatments, rather than serving as a standalone approach. In addition, the interspecies differences in thermogenic capacity and responsiveness further explain the gap between preclinical efficacy and clinical outcomes, underscoring the need for realistic expectations when translating browning-based interventions to human MASLD.

Moving forward, research must shift toward bridging mechanistic insights with clinically meaningful outcomes. Future work includes identifying patients with preserved thermogenic capacity, optimizing combination therapies, and developing strategies that enable sustained and safe activation of browning in human populations. A particularly important priority is establishing whether adipose browning is a causal determinant of hepatic and metabolic improvement or primarily a biomarker of preserved metabolic health; while a deeper investigation into inter-individual variability influenced by age, genetics, and environmental factors are also needed. To address these questions, future studies should incorporate longitudinal cohort designs that track BAT activity and liver outcomes over time, Mendelian randomization analyses leveraging genetic determinants of thermogenic capacity, and controlled interventional studies that directly modulate BAT activity while prospectively evaluating hepatic steatosis, inflammation, and fibrosis. Such approaches will be essential for determining whether adipose browning represents a true therapeutic target in MASLD or serves primarily as an indicator of broader metabolic resilience [93, 99, 100].

Advances in precision medicine offer opportunities to target key regulators such as PPARs, FGF21, and mitochondrial pathways, while optimizing lifestyle-based strategies, including exercise, dietary modulation, and controlled cold exposure, remain essential for scalable interventions. Additionally, integrating multi-omics approaches and systems biology frameworks may clarify unresolved aspects of adipose–liver crosstalk and identify novel therapeutic targets.

From a preclinical perspective, future MASLD models should be leveraged to dissect depot-specific contributions of adipose tissue to disease progression, particularly the distinct roles of visceral depots such as eWAT, thermogenically responsive subcutaneous depots such as iWAT, and classical BAT [24, 25]. Such models may help clarify how individual adipose depots differentially influence hepatic steatosis, inflammation, and fibrosis while identifying browning-associated molecular signatures and batokine profiles predictive of treatment response [20, 22]. In addition, combination studies evaluating browning-targeted interventions alongside established metabolic therapies, including THR-β agonists, PPAR modulators, and weight-loss strategies, may provide a more realistic assessment of therapeutic efficacy in metabolically complex disease settings [113, 130, 131].

From a clinical perspective, adipose browning and batokine biology may be increasingly integrated into MASLD drug-development pipelines, particularly in combination with emerging therapies such as FGF21 analogs, incretin-based therapies, and PPAR-targeted agents [113–115]. Browning-related biomarkers, including BAT activity assessed by PET/CT imaging as well as circulating mediators such as FGF21, Nrg4, and irisin [120, 177, 198], may provide useful tools for patient stratification, pharmacodynamic assessment, and treatment monitoring in future clinical trials. However, given the limited thermogenic capacity observed in many individuals with obesity and advanced MASLD, adipose browning is most realistically viewed as an upstream metabolic modulator that is likely to augment the benefits of weight loss and systemic metabolic therapies rather than function as a standalone treatment strategy. Ultimately, translating adipose browning into a viable strategy for MASLD will require coordinated efforts across basic science, clinical research, and public health, with careful consideration of population heterogeneity and real-world feasibility.

## Key References

- Fisher, F.M., et al., FGF21 regulates PGC-1α and browning of white adipose tissues in adaptive thermogenesis. *Genes Dev*, 26(3): p. 271–281. (2012).
  - *This foundational mechanistic study identifies FGF21 as a central regulator of adipose browning, demonstrating its role in inducing thermogenic gene programs *via* PGC-1α. It provides critical insight into how endocrine signaling enhances energy expenditure and underpins adipose–liver metabolic crosstalk.*
- Chondronikola, M., et al., Brown adipose tissue improves whole-body glucose homeostasis and insulin sensitivity in humans. *Diabetes*, 63(12): p. 4089–4099. (2014).
  - *This landmark human study demonstrates that BAT activation improves insulin sensitivity, glucose disposal, and energy expenditure. It provides direct clinical evidence supporting BAT as a metabolically relevant tissue in adults and a therapeutic target for metabolic disease.*
- Wang, G.X., et al., The brown fat–enriched secreted factor Nrg4 preserves metabolic homeostasis through attenuation of hepatic lipogenesis. *Nat Med*, 20(12): p. 1436–1443. (2014).
  - *This pivotal study identifies Nrg4 as a BAT-derived endocrine factor that suppresses hepatic lipogenesis and protects against steatosis, establishing a direct adipose–liver signaling axis central to MASLD pathophysiology.*
- Harrison, S.A., et al., Efruxifermin in non-alcoholic steatohepatitis: a randomized, double-blind, placebo-controlled phase 2a trial. *Nat Med*, 27(7): p. 1262–1271. (2021).
  - *This clinical trial demonstrates that FGF21 analog therapy significantly reduces hepatic fat and improves metabolic parameters in nonalcoholic steatohepatitis, providing strong translational validation of adipose–liver endocrine pathways.*
- Chondronikola, M., et al., Brown adipose tissue activation is linked to distinct systemic effects on lipid metabolism in humans. *Cell Metab*, 23(6): p. 1200–1206. (2016).
  - *This study shows that BAT activation enhances lipid turnover and fatty acid oxidation, supporting the concept that BAT acts as a metabolic sink that reduces lipid availability for hepatic accumulation.*
- Chen, M., et al., Exercise-induced adipokine Nrg4 alleviates MASLD by disrupting hepatic cGAS–STING signaling. *Cell Rep*, 44(2): 115251. (2025).
  - *This recent study demonstrates that Nrg4 mediates exercise-induced protection against MASLD by suppressing hepatic inflammatory signaling, highlighting the importance of adipose-derived factors in disease modulation.*

## Data Availability

No datasets were generated or analysed during the current study.
